# Comparison of Ketamine-Propofol and Ketamine-Thiopental on Bispectral Index Values during Monitored Anesthesia Care (MAC) in Minor Traumatic Orthopedic Surgery; A Randomized, Double-Blind, Clinical Trial

**DOI:** 10.29252/beat-070205

**Published:** 2019-04

**Authors:** Afsaneh Nowroozi, Hanieh Kianipour, Houshang Taleby, Bijan Yazdi

**Affiliations:** 1 *Department of Anesthesiology, Arak University of Medical Sciences, Arak Iran*

**Keywords:** Monitored anesthesia care, Ketamin, Propofol, Thiopantal sodium

## Abstract

**Objective:**

To compare the effects of ketamine-propofol and ketamine-thiopental on bispectral index values during monitored anesthesia care in minor orthopedic surgeries.

**Methods:**

This randomized double-blind clinical trial was performed on 90 patients undergoing minor orthopedic surgeries. Participants were randomly allocated to either groups of propofol or thiopental. Bispectral index (BIS), non-invasive arterial blood pressure, SpO2, and electrocardiogram were monitored every 5 minutes. Patients in propofol group received a bolus dose of 0.5 mg/kg ketamine, plus 0.5 mg/kg propofol. In thiopental group, patients received a bolus dose of 0.5 mg/kg ketamine, plus 50-75 mg thiopental. After the surgery, recovery duration, patients’ pain score (VAS) and any intra-operative recall or awareness were recorded. Statistical analysis was performed using SPSS version 19.0

**Results:**

BIS was lower in ketamin-propofol group (*p*< 0.001). Mean arterial blood pressure, heart rate and O2 saturation showed a significant difference between two groups (*p*< 0.001), which were lower in ketamin–propofol group. VAS score was higher in ketamin-thiopental group (*p*< 0.001). Both groups were similar in intra-operative recall/awareness.

**Conclusion:**

Ketamine-propofol combination deliver a better control over monitored anesthesia during surgery, providing lower BIS, higher O2 saturation and lower heart rate and arterial blood pressure in patients undergoing minor traumatic orthopedic surgeries.

**Clinical trial registration:**

IRCT6N 2016032320258

## Introduction

Monitored anesthesia care (MAC) is a planned procedure during which the patients undergo local anesthesia together with analgesic and sedative procedures, in order to achieve a safe sedation together with anxiety and pain control [1], during which the patient is at a level of sedation that can respond to verbal or tactile stimulus, meanwhile the airways are maintained [2]. The ideal agent is an anesthetic which is adequately sedating, characterized by high efficiency and ultra-fast recovery, since the context-sensitive half-time (CSHT) is very short: approximately 3 minutes [1] without causing toxicity [3]. Moreover, MAC allows the patients to be discharged very soon. Ketamine is a strong N-methyl D aspartate receptor antagonist which produce anesthesia, analgesia, and some degrees of amnesia. It rapidly distributes through CNS via the blood-brain barrier due to its high lipid solubility and induces anesthesia within 45 to 60 seconds. Unlike other anesthetic agents, this medication also maintains airway and airway reflux, but high dose or bolus injection can cause apnea. Also, ketamine can cause hypertension, nausea, dysphagia and illusions [4,5]. 

Another anesthetic agent with sedative effects is propofol, which is used extensively because of its rapid anesthesia-induction and recovery time; but due to its lack of analgesic effect, higher doses should be used in surgeries requiring analgesia [6]. Propofol-induced anesthesia that is too deep is associated with cardiovascular and respiratory system dysfunction, while light anesthesia can lead to episodes of intra-operative awareness [7,8]. The main concern of propofol-induced hypnosis is providing proper sedation depth, while responding to verbal stimulus, without intra-operative awareness or recall. The incidence of intra-operative awareness in healthy patients is about 0.1% and can increase to 1 to 1.5% in high-risk populations [9]. BIS monitor is the first quantitative EEG index used in clinical practice as a monitor to assess the depth of anesthesia. It consists of a sensor, a digital signal converter, and a monitor. The sensor is placed on the patient’s forehead to pick up the electrical signals from the cerebral cortex and transfer them to the digital signal converter [10]. This method makes it possible to assess the depth of anesthesia and titrating the administration of anesthetic agents, thereby assures accurate dosage and reduces unwanted adverse effects. BIS-guided anesthesia also lowers the incidence and severity of postoperative nausea and vomiting and improves the time to recovery and home readiness which is more economical [11]. Single-drug sedation can be attained using sedatives such as propofol, methohexital, midazolam, etomidate, ketamine, thiopental or primary classes of analgesics, such as narcotics. Since there is no ideal agent with all these features, higher doses of sedatives should be used which in turn increases the rate of adverse effects. To avoid these complications, a combination of several agents may be used at a lower dose [12] which has been shown in numerous studies to be safe and effective, without posing a risk of complications. This study’s purpose was to compare the effects of ketamine-propofol and ketamine-thiopental on bispectral index values during monitored anesthesia care in minor orthopedic surgeries. 

## Materials and Methods

 *Study population*

  This double-blind parallel-group randomized clinical trial was designed and conducted in an academic hospital (Arak, Iran) during 2015. Before recruitment of the first subject, Study protocol was approved by local ethics committee of Arak University of Medical Sciences (Registration ID: IR.ARAKMU.REC42.1394) and was registered in Iranian Registry of Clinical Trials (IRCT6N 2016032320258; www.irct.ir).The study has been performed in accordance with the ethical standards of the 1964 Declaration of Helsinki. All patients signed the informed consent forms prior to recruitment in the study. Study population were all patients referred to our university hospital for minor orthopedic surgeries. Inclusion criteria were age between 18-65 years, ASA class of I-II, and less than 30 minutes of surgery duration. Exclusion criteria were unwilling to participate in the study, consumption of sedatives or hypnotic drugs, history of psychological illness or drug abuse, and pre-existing allergies especially to anesthetic agents. 

 *Randomization and intervention*

  Participants were randomly allocated to either groups of propofol or thiopental. Baseline and demographic data were documented in patients’ profile. Upon arriving to the operation room, a 20G venous canula, was inserted and patients received NS solution 5 ml/kg plus 10 mg metoclopramide. The sensors were placed on the patient’s forehead and bispectral index (BIS) was monitored with a BIS XP (Danmeter A/S Kildemosevej 13, DK-5000 Odense C, Denmark). Non-invasive arterial blood pressure, SpO2, and electrocardiogram were monitored routinely. Patients in propofol group received a bolus dose of 0.5 mg/kg ketamine (150077mfd - Sterop Belgium), plus 0.5 mg/kg propofol (Dongkook Pharm). In thiopental group, patients received a bolus dose of 0.5 mg/kg ketamine (150077mfd - Sterop Belgium), plus 50-75 mg thiopental (Inresa Arzneimittel).

 *Study protocol *

 Every 5 minutes since initiation of surgery, vital signs and of anesthetic depth was recorded. When surgery was done, patients were asked to express their pain score according to visual analogue scale (VAS). Patients were also questioned whether they could recall their surroundings, or an event related to the surgery. Recovery duration (i.e. time from arriving to the recovery room till full consciousness) were recorded as well. To ensure blindness, data gathering was performed by an anesthesiologist unaware of patients’ allocated group.

 *Statistical analysis*

  Sample size was determined to be 45 per group, assuming an α-error of 0.05, power of 80%, and drop-out rate of 10%. Sampling was performed using simple random sampling method. When the study was completed, statistical analysis was performed using SPSS version 19.0 for Windows (SPSS Inc, Chicago, IL, USA). Data are expressed as mean ± standard deviation (SD). One-way analysis of variance and two-sample *t*-test were used to compare values for normality. Proportions were compared using chi-square test. A two-sided p-value of less than 0.05 was considered statistically significant.

## Results

**Fig. 1:**
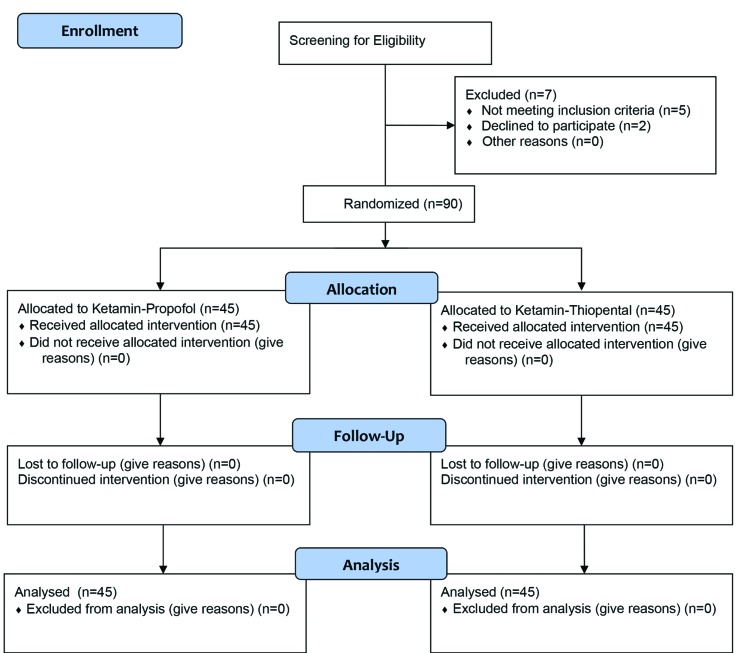
The CONSORT flow diagram of the study.

**Fig. 2:**
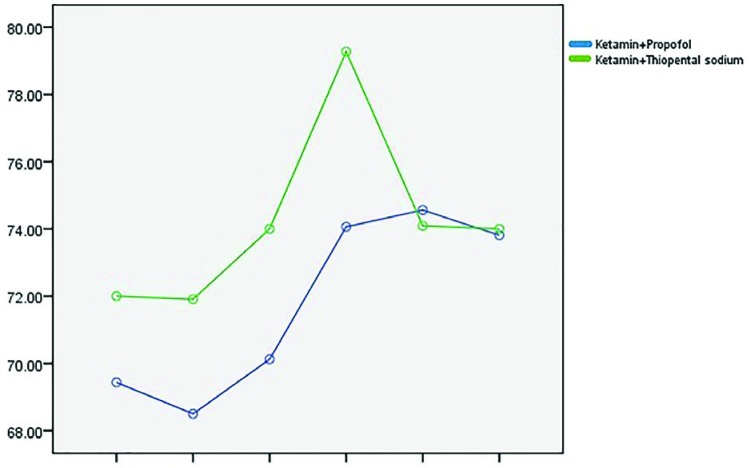
Comparison of bispectral index values (BIS) between the two groups in different time points.

Ninety participants including 21 women (23.3%), and 69 men (76.7%) undergoing minor orthopedic surgeries completed the study (Table 1). The study flow diagram is demonstrated in Figure 1. In propofol group, 36 patients were men (80%) and 9 were women (20%). In thiopental group, 33 patients were men (73.3%) and 12 were women (26.7%). Mean age of the participants was 30.55±1.10 years. Mean surgery duration was 23.61±4.68 minutes. Age, gender, surgery duration and baseline VAS & BIS showed no statistically significant difference between the two groups (Table 1). Recovery duration showed significant difference between the two groups and it was lower in propofol group (14.22±4.38 minutes in propofol group VS 21.88±5.03 minutes in thiopental group) (*p*=0.0001). BIS showed significant difference between the two groups and in different time points: 5, 10, 15 & 20 minutes after initiation of surgery, BIS was lowered in propofol group (*p*=0.0003), although in 25th and 30th minutes, no statistically significant difference was noted (Figure 2).

**Table 1:** The baseline characteristics of the patients included in the current study.

	**Ketamin-Propofol (n=45)**	**Ketamin-Thiopental (n=45)**	**p-value **
**Age (years)**	30.97 ± 1.14	30.13 ± 1.7	0.078
**Gender **			
**Men (%)**	36 (80%)	33 (73.3%)	0.082
**Women (%)**	9 (20%)	12 (26.7%)	0.151
**Duration of surgery (min)**	24.00 ± 4.95	23.22 ± 4.41	0.093
**Mean Baseline VAS**	7	7	0.641
**Mean Recovery time (min)**	14.22 ± 4.38	21.88 ± 5.03	0.001

**Fig. 3:**
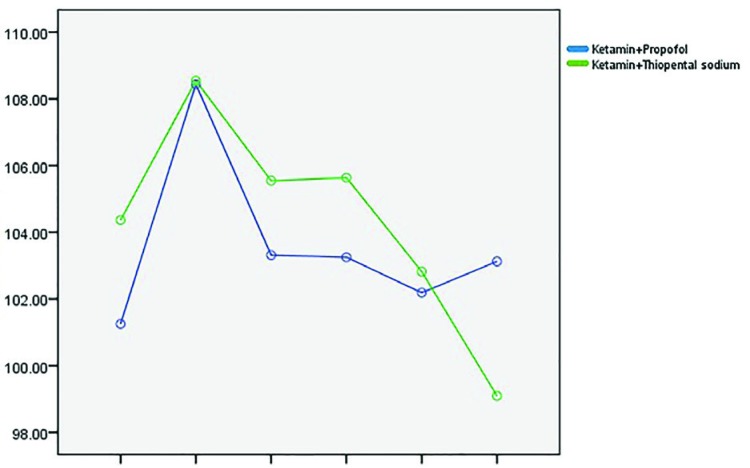
Comparison of mean arterial pressure (MAP) between the two groups in different time points.

**Fig. 4:**
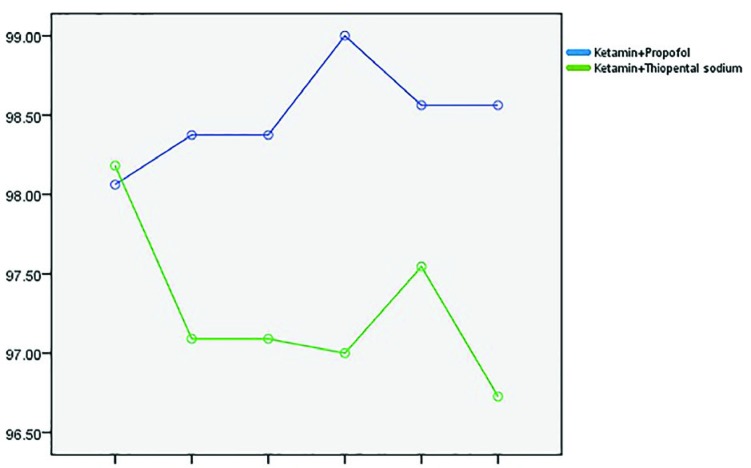
Comparison of O2 saturation between the two groups in different time points.

**Fig. 5:**
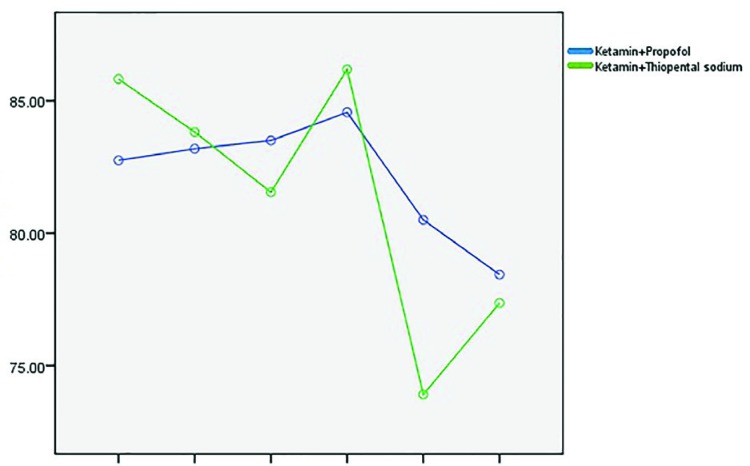
Comparison of heart rate (HR) between the two groups in different time points.

Mean arterial blood pressure (MAP) between the two groups were different in 5th minutes after the surgery commencement, in thiopental group MAP was higher and the difference was statistically significant (*p*=0.037). Although in other time points no significant difference was noted (Figure 3). O2 saturation showed significant difference between the two groups and in different time points: 10, 15 and 20 minutes after initiation of surgery, O2 sat was lowered in thiopental group (*p*=0.002), although in other time points, no statistically significant difference was noted (*p*=0.241), (Figure 4). Five and 10 minutes after initiation of surgery, heart rate showed significant difference between the two groups and HR was lowered in propofol group (*p*=0.001). In other time points, no statistically significant difference was noted (*p*=0.822), (Figure 5).

## Discussion

The purpose of this study was to compare the effects of ketamine-propofol and ketamine-thiopental on bispectral index values during monitored anesthesia care (MAC). The study was a double blind clinical trial on 90 patients undergoing minor orthopedic surgery. Regarding our findings, it seems that ketamine-propofol combination deliver a better control over monitored anesthesia during surgery, providing lower BIS, higher O2 saturation and lower heart rate and arterial blood pressure.

In 2011, and in a randomized clinical trial, Sorensen et al. evaluated onset time and hemodynamic response after thiopental vs. propofol in the elderly. Their findings showed that time to BIS <50 was significantly shorter in patients receiving thiopental, where onset time was 52 s (median value) compared with 65 s in the propofol group (*p*=0.01). Mean arterial pressure decreased 25.6 mmHg in the propofol group and 15.6 mmHg in the thiopental group (*p*=0.003) within 120 s. Heart rate decreased 9.1 b.p.m. within 120 s in the patients receiving propofol compared with a decrease of 5.1 b.p.m. in patients receiving thiopental (*p*=0.04). They concluded that Thiopental was found to have a faster onset than propofol in elderly surgical patients which is not consistent with findings from our trial [13].

 In a prospective, double-blinded, randomized trial by Nejati *et al*. in 2011, which was performed on emergency department (ED) patients requiring procedural sedation and analgesia, researchers compared the ketamine/propofol combination with the midazolam/fentanyl combination. The median starting doses were 0.75 mg/kg of both ketamine and propofol, 0.04 mg/kg midazolam, and 2 μg/kg fentanyl. There were no significant differences in sedation time between the groups. Perceived pain in the ketofol group, as measured by the Visual Analog Scale (VAS), was significantly lower than in the MF group (*p*<0.001). They concluded that ketamine/propofol combination provides adequate sedation and analgesia for painful procedures and appears to be a safe and useful technique in the ED [14]. The results of this study are in accordance with our findings. Henry *et al*. in 2011, compared the frequency of respiratory depression, sedation quality and total propofol dose during emergency department procedural sedation with ketamine plus propofol versus propofol alone. The incidence of respiratory depression was similar in both groups and there was no statistically significant difference. With ketamine/propofol compared with propofol alone, less propofol was administered, and there was a trend toward better sedation quality. They concluded that the combination of ketamine and propofol Compared with procedural sedation with propofol alone, did not reduce the incidence of respiratory depression but resulted in greater provider satisfaction, less propofol administration, and perhaps better sedation quality [15] which are also observed in our study. Thomas et al in 2011 discussed the safety of using ketamine-propofol combinations as an alternative to using either agent alone for procedural sedation. The combined use of ketamine and propofol is a reasonable alternative to propofol alone for procedural sedation in patients at higher risk for respiratory depression or hypotension [16], which is in line with our study.

Yuri *et al*., in 2011 compared the recovery time, total sedation time, and the adverse events of procedural sedation and analgesia induced with propofol as compared with midazolam/ketamine. They hypothesized that sedation with propofol as compared with midazolam/ketamine will save time in the emergency department. The average recovery time was 7.8±3.7 minutes following sedation with propofol, compared with 30.7±10.1 minutes following sedation with midazolam/ketamine (*p*<0.001). The average total sedation time was 16.2±3.8 minutes for the propofol group, compared with 41.6±10.7 minutes for the midazolam/ketamine group (*p*<0.001). The overall rate of respiratory and hemodynamic adverse events was 20% for the propofol group and 10% for the midazolam/ketamine group. They concluded that use of propofol for an orthopedic procedure requiring sedation in the emergency department expedites patient management and saves time in comparison with the use of midazolam/ketamine [17]. In our study, recovery was also lower in the ketamine-propofol group, which is consistent with the results of Yuri *et al*. 

Rabiee *et al*. in 2011 conducted a double blind clinical trial and compared sodium thiopental and propofol as induction agents in depth of anesthesia and hemodynamic variations in mothers and APGAR score of neonates. BIS values in different times, was similar and less than 60 (*p*=0.637). Maternal mean arterial pressure (*p*=0.630) and heart rate (*p*=0.623) and neonatal APGAR score in first minute (*p*=0.105) and fifth minute (*p*=0.185) were not significantly different. They concluded that effect of sodium thiopental and propofol on depth of anesthesia and hemodynamic variables of mothers as well as neonatal APGAR scores was similar and propofol can be used as an appropriate alternative for sodium thiopental in induction of anesthesia for cesarean section [18]. Meanwhile, in our study, Ketamine-Propofol group showed a lower BIS than Ketamine-Thiopental sodium group. There was a significant difference in the type of surgery, which can ne pertained to surgical type: Cesarean section in Rabi'i *et al*., and minor orthopedic surgeries in our study. On the other hand, both genders were included in our study, but study population in Rabiee *et al*. was women only. The sample size was also higher in our study.

In a prospective, randomized case series of patients undergoing procedural sedation for fracture manipulation, Phillips et al evaluated the comparative effectiveness and safety of propofol versus propofol/ketamine combination for procedural sedation using bispectral index monitoring for measuring depth of sedation. Patients were randomized to a propofol (P) group with a target dose of 0.5 to 1.5 mg/kg or a propofol/ketamine (P/K) group with a target dose of both ketamine and propofol of 0.75 mg/kg. Procedural success, bispectral index (BIS) score, adverse effects, recovery time, and vital signs were measured. The P/K group experienced a smaller decline in systolic blood pressure (1.6% versus 12.5%) and BIS score at goal sedation (77 versus 61), a smaller difference between baseline and goal sedation BIS score (18.78±10 versus 34.64±11) and a lower mean propofol dose (92.5±58 versus 177.27±11 mg). No patient in either group experienced respiratory depression or required any intervention. According to their findings, the combination of propofol and ketamine provides an attractive combination for procedural sedation in the emergency department. Compared to propofol alone, "ketofol" resulted in less hypotension, better sedation, and enhanced patient comfort and safety [4].

The current study was conducted in a single academic center, and generalizability of the results require a multicenter study. Further extensive randomized, prospective studies with larger sample sizes comparing ketamine and propofol with other common agents could further document the safety, efficacy, and effectiveness of the ketamine and propofol combination for PSA along with any rare but possible adverse effect.

In conclusion, ketamine-propofol combination deliver a better control over monitored anesthesia during surgery, providing lower BIS, higher O2 saturation and lower heart rate and arterial blood pressure.

## References

[1] Pergolizzi JV Jr, Gan TJ, Plavin S, Labhsetwar S, Taylor R (2011). Perspectives on the role of fospropofol in the monitored anesthesia care setting. Anesthesiol Res Pract.

[2] American Society of Anesthesiologists (2002). Task Force on Sedation and Analgesia by Non-Anesthesiologists Practice guidelines for sedation and analgesia by non-anesthesiologists. Anesthesiology.

[3] Schneider MS, Coates WC (1996). Use of ultrashort-acting hypnotic agents in emergency departments. West J Med.

[4] Phillips W, Anderson A, Rosengreen M, Johnson J, Halpin J (2010). Propofol versus propofol/ketamine for brief painful procedures in the emergency department: clinical and bispectral index scale comparison. J Pain Palliat Care Pharmacother.

[5] Godambe SA, Elliot V, Matheny D, Pershad J (2003). Comparison of propofol/fentanyl versus ketamine/midazolam for brief orthopedic procedural sedation in a pediatric emergency department. Pediatrics.

[6] Akcaboy ZN, Akcaboy EY, Albayrak D, Altinoren B, Dikmen B, Gogus N (2006). Can remifentanil be a better choice than propofol for colonoscopy during monitored anesthesia care?. Acta Anaesthesiol Scand.

[7] Smith I, Monk TG, White PF, Ding Y (1994). Propofol infusion during regional anesthesia: sedative, amnestic, and anxiolytic properties. Anesth Analg.

[8] Taylor E, Ghouri AF, White PF (1992). Midazolam in combination with propofol for sedation during local anesthesia. J Clin Anesth.

[9] Miller RD, Eriksson LI, Fleisher LA, Wiener-Kronish JP, Cohen NH, Young WL ( 2014). Miller's Anesthesia E-Book: Elsevier Health Sciences.

[10] March PA, Muir WW (2005). Bispectral analysis of the electroencephalogram: a review of its development and use in anesthesia. Vet Anaesth Analg.

[11] Davidson A (2004). The correlation between bispectral index and airway reflexes with sevoflurane and halothane anaesthesia. Paediatr Anaesth.

[12] Black E, Campbell SG, Magee K, Zed PJ (2013). Propofol for procedural sedation in the emergency department: a qualitative systematic review. Ann Pharmacother.

[13] Sørensen MK, Dolven TL, Rasmussen LS (2011). Onset time and haemodynamic response after thiopental vs propofol in the elderly: a randomized trial. Acta Anaesthesiol Scand.

[14] Nejati A, Moharari RS, Ashraf H, Labaf A, Golshani K (2011). Ketamine/propofol versus midazolam/fentanyl for procedural sedation and analgesia in the emergency department: a randomized, prospective, double-blind trial. Acad Emerg Med.

[15] David H, Shipp J (2011). A randomized controlled trial of ketamine/propofol versus propofol alone for emergency department procedural sedation. Ann Emerg Med.

[16] Thomas MC, Jennett-Reznek AM, Patanwala AE (2011). Combination of ketamine and propofol versus either agent alone for procedural sedation in the emergency department. Am J Health Syst Pharm.

[17] Uri O, Behrbalk E, Haim A, Kaufman E, Halpern P (2011). Procedural sedation with propofol for painful orthopaedic manipulation in the emergency department expedites patient management compared with a midazolam/ketamine regimen: a randomized prospective study. J Bone Joint Surg Am.

[18] Rabiee S, Alijanpour E, Naziri F, Alreza H, Esmaeili V (2012). A Comparison of depth of anesthesia and hemodynamic variables with sodium thiopental and propofol as induction agents for cesarean section. Journal of Babol University of Medical Sciences (JBUMS).

